# A Review on Toxic and Harmful Algae in Greek Coastal Waters (E. Mediterranean Sea)

**DOI:** 10.3390/toxins2051019

**Published:** 2010-05-11

**Authors:** Lydia Ignatiades, Olympia Gotsis-Skretas

**Affiliations:** 1 National Center of Scientific Research “Demokritos”, Institute of Biology, Aghia Paraskevi, 15310 Athens, Greece; 2 Hellenic Center for Marine Research, Institute of Oceanography, 19013 Anavyssos, Greece; Email: ogotsis@ath.hcmr.gr

**Keywords:** : harmful algae, Aegean Sea, Ionian Sea

## Abstract

The Greek coastal waters are subjected to harmful algal bloom (HAB) phenomena due to the occurrence of species characterized as toxic (TX), potentially toxic (PT), and non-toxic, high biomass (HB) producers causing harm at multiple levels. The total number of (TX), (PT) and (HB) algae reported in this work are 61, but only 16 species have been associated with the occurrence of important HABs causing damage in the marine biota and the water quality. These phenomena are sporadic in time, space and recurrence of the causative species, and are related to the anthropogenically-induced eutrophication conditions prevailing in the investigated areas.

## 1. Introduction

The coastline (18,000 km) of the Greek mainland is located in the Eastern Mediterranean Sea, it is surrounded by the Aegean, Ionian and Cretan Seas and its morphological regime shows a variety of gulfs and semi-enclosed gulfs. All these basins are eutrophic [1] since they receive the waters and fertile material from large rivers and/or smaller water outfalls derived from agricultural and industrial activities. 

Eutrophication triggers various physical and chemical changes in the marine environment and exerts a pressure on algal populations, allowing the intensive growth of certain harmful-toxin producing species or nuisance blooms that may create problems in the structure of the ecosystem and public health. These blooms are collectively called Harmful Algal Blooms (HABs). The greatest number of toxic species is found among dinoflagellates, but evidence has been provided for several species of other taxa (diatoms, flagellates, cyanobacteria, prymnesiophytes, rhaphidophytes) suggesting that they belong in this category [2,3,4]. 

Concern about harmful algae in Greek coastal waters has been growing since the late 1970s, when the first symptoms of “fish kills” due to the increased anthropogenic effects led to the fact that HABs-often quoted as the phenomenon of red tides-acquired the attention of scientists and the public. Since then, routine records of phytoplankton samples from almost all major gulfs along the Greek coastline during the last 30 years have revealed the presence of toxic and potentially toxic algae (those producing and/or potentially producing toxins) and non-toxic, high biomass producing species (non-toxic producers, but causing harmful blooms at multiple levels), although their destructive effects were occasional.

The European Commission has funded a number of projects such as EUROHAB (European Initiative on Harmful Algal Blooms) to generate the required research to better manage the effects of toxic/harmful marine microalgae that have caused problems in European marine waters [5]. This paper is the first comprehensive presentation of these species in the Eastern Mediterranean Sea, based on a synopsis of all published information for the period 1977-2009. 

## 2. Sampling Areas and Data Collection

**Figure 1:**
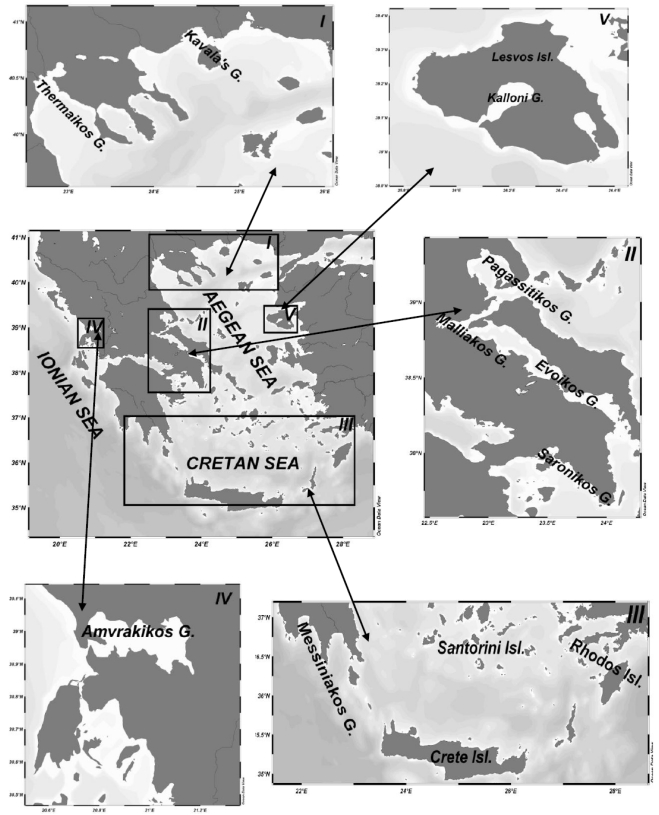
Location of the investigated Gulfs on the map of Greece.

The investigated area (Figure 1) is located in the Eastern Mediterranean and presents the sampling regions along the coastlines of the North Aegean Sea (I), the Western Aegean Sea (II), the Southern Aegean Sea (III), the Ionian Sea (IV) and the Mytilini Island, Eastern Aegean Sea (V). These sites include nine major Gulfs (a: Thermaikos; b: Kavalas; c: Pagassitikos; d: Malliakos; e: Evoikos; f: Saronikos; g: Messiniakos; h: Amvrakikos and i: Kalloni), as well as harbors, docks and marinas. 

The collection of data covers the period 1977-2008. The methodology of sampling, preservation of samples, quantitative-qualitative analysis and the toxicity detection/evaluation of each one of the phytoplanktonic species under investigation are given in the literature cited in Table 1. The characterization of species as toxic (TX), potentially toxic (PT) and high biomass (HB) harmful blooms in this work was based on publications providing comprehensive descriptions of the current status of knowledge in the field as well as the IOC-UNESCO Taxonomic Reference List of Harmful Micro Algae [4]. The specifications of toxins were also determined from the literature. 

## 3. Results and Discussion

A traditional system has been adopted for the eukaryotic species taxonomy [6]. Cyanobacteria are prokaryotes that may create problems producing diverse neurotoxins hazardous for human health; they have been classified among the HAB species [7] and are therefore included here. The majority of species are autotrophic (photosynthetic algae), but certain species (mostly dinoflagellates) are heterotrophic (feeding on particulate or dissolved organic matter) and their mode of nutrition (phagotrophy, osmotrophy) has been also taken into consideration [8]. It is interesting to notice that species of the same family differ in toxic properties. 

### 3.1. Taxonomy and toxic properties of detected HAB species in Greek coastal waters

#### 3.1.1. Class Bacillariophyceae (Diatoms)

##### 3.1.1.1. Order Thalassiophysales

Family Catenulaceae. A strain of the species *Amphora coffeaeformis *(from Canada) was found to produce Domoic acid. Other strains examined so far were non-toxic. However, the precise identification of the Canadian strain has been questioned [4,9].It is also a mucilage producer [10].

##### 3.1.1.2. Order Bacillarialles

Family Bacillariaceae. The five species of this family are Domoic acid producers: Pseudo-nitzschia delicatissima [11], Pseudo-nitzschia pseudodelicatissima [12], Pseudo-nitzschia seriata [13], Pseudo-nitzschia pungens [14] and Pseudo-nitzschia calliantha [15]. 

#### 3.1.2. Class Dinophyceae (Dinoflagellates)

There are five important orders of Dinophyceae identified and presented in this work: Peridiniales, Prorocentrales, Dinophysiales, Gymnodiniales, and Noctilucales.

##### 3.1.2.1. Order Peridiniales

Family Goniodomataceae. This family comprises six species of the genus *Alexandrium * and one of the genus *Gambierdiscus* that are among the well known harmful algae. *A. catenella *is a producer of c1-c4 toxins, Saxitoxins and Gonyautoxins [16,17]. *A. tamarense*, *A. minutum *and *A. taylori *produce Gonyautoxins [18,19]. *A. balechii and A. insuetum* have been characterized in the literature as species of unknown toxicity, but they have been associated with harmful algal blooms [14,20] and *Gambierdiscus *sp. is known to be toxic producing Ciguatoxin and Maitotoxine [21].

Family Ostreophidaceae. *Coolia monotis *produces Cooliatoxin, an analog of Yessotoxin [22]. 

Family Heterocapsaceae. *Heterocapsa circularisquama *produces the photosensitizing hemolytic toxins H2-a, H3-a [23]. 

Family Ostreopsidaceae. The two toxic species of the genus *Ostreopsis *are *O. ovata* producing putative Palytoxin and Ovatoxin compounds and *O. siamensis*, putative Palytoxin [24,25,26].

Family Gonyaulacaceae. *Protoceratium reticulatum* is a species known as a Yessotoxin toxin producer [27]. *Scrippsiella trochoidea* is a bloom forming species of unknown toxicity [28].

Family Protoperidiniaceae. Two species of this family have been recorded, *Diplopsalis lenticula*, a bloom forming species [29] of unknown toxicity and *Protoperidinium crassipes, *producingAzaspiracid toxins [30].

Family Peridiniaceae. *Peridinium quinquecorne* is a bloom forming species [31].

Family Ceratiaceae. The four species of the genus *Ceratium*, *C. furca*, *C. fusus*, *C. lineatum*, and *C. tripos* occasionally form non-toxic blooms [32] that may cause discoloration of the water and undesirable aesthetic symptoms, but without toxic signs [33,34,35,36]. 

##### 3.1.2.2. Order Prorocentrales

Family Prorocentraceae. All species of this family are in the genus *Prorocentrum.* The four toxic species are: *P. borbonicum*, producing Borbotoxins [37], *P. levis* and *P. lima*, producing Okadaic acid and Dinophysistoxins [38,39], and the Okadaic acid producer *P. rhathymum* [40]. Species associated with high biomass harmful blooms are: *P. arcuatum* [41], *P. obtusidens* [43], *P. redfeldii *[43], *P. micans* [44], *P. minimum* [45], *P. dentatum *[46] and *P. emarginatum* [47]. 

##### 3.1.2.3. Order Dinophysiales

Family Dinophysiaceae. All species of this family representing the genus *Dinophysis* are toxic. *D. sacculus *produces Okadaic acid [48]; *D. tripos *and *D. rotundata*, Dynophysistoxin [48,49]; *D. acuminata *and D. *acuta*, Okadaic acid/Dynophysistoxin [48]; *D. fortii*, Okadaic acid/Dynophysistoxin/Palytoxin [48]; *D. caudata,* Okadaic acid/Palytoxin [48]. 

##### 3.1.2.4. Order Gymnodiniales

Family Gymnodiniaceae. All species of this family are toxic except *Gyrodinium impudicum*, a non-toxic, bloom forming species [50]. *Amphidinium carterae, *is a producer of the Maitotoxin [51] and *G. aureolum* produces 1-acyl-3-digalactosylglycerol and octadecapentaenoic acid [52]. One of the two toxic species of the genus *Karenia, K.brevis* produces Polyether Neurotoxins called Brevetoxins [53] and *K. mikimotoi* Gymnocin-A [54]. The species *Gymnodinium catenatum* produces Gonyautoxins and Saxitoxin [55], whereas *Karlodinium veneficum*, Karlotoxins [56]. 

##### 3.1.2.5. Order Noctilucales

Family Noctilucaceae. *Noctiluca scintillans* is the single species of this family. It is a non-toxic bloom forming species [57] responsible for harmful outbursts (water discoloration, anoxic events). 

#### 3.1.3. Class Prymnesiophyceae (Haptophytes)

##### 3.1.3.1. Order Phaeocystales

Family Phaeocystaceae. *Phaeocystis puchetii*: toxic species producing polyunsaturated aldehyde [58].

##### 3.1.3.2. Order Prymnesiales

Family Prymnesiaceae. *Prymnesium parvum*: toxic species producing Prymnesins [59].

#### 3.1.4. Class Rhaphidophyceae (Chloromonadophytes)

Order Chattonellalles

Family Chattonellaceae. Both species of this family *Chattonella globosa* and *C.verucolosa *are unknown toxicity high biomass forming species [60]. 

#### 3.1.5. Class Cyanophyceae (Cyanobacteria)

##### 3.1.5.1. Order Chroococales

Family Chroococaceae. The species *Microcystis aeruginosa* produces the toxin Microcystin-LR [61], and the species *Chroococcus gelatinosus* and *Synechocystis sallensis* are bloom forming species [62].

##### 3.1.5.2. Order Nostocalles

Family Oscillatoriaceae. *Lyngbya agardhii* is a high biomass forming species [62] and* Trichodesmium erythraeum* produces Saxitoxin [63].

Table 1 presents alphabetically the list of species, their toxic properties and the area of their occurrence given in the literature. 

**Table 1:** Toxic (TX), potentially toxic (PT) and high biomass (HB) nuisance species in Greek coastal waters. Shaded rows demarcate species that have caused toxic events.

Species	Toxins	Category	Area	Source
**Diatoms**				
*Amphora coffeaeformis *(C. Agardh) Kützing	Domoic acid	(PT)	V	[29]
*Pseudo-nitzschia calliantha*, Lundholm, Moestrup et Hasle	Domoic acid	(PT)	V	[29]
*Pseudo-nitzschia delicatissima * (Cleve) Heiden	Domoic acid	(PT)	I, II, III	[73,77]
*Pseudo-nitschia pseudodelicatissima* (Hasle) Hasle	Domoic acid	(PT)	I, II, IV, V	[43,78,79]
*Pseudo-nitzschia pungens* (Grunow ex Cleve) Hasle	Domoic acid	(PT)	I, II, III, IV, V	[29,43,73]
*Pseudo-nitzschia seriata * (Cleve) H. Peragallo	Domoic acid	(PT)	I, II, III, V	[78,80,81]
**Dinoflagellates**				
*Alexandrium balechii* (Steidinger) Balech	Unknown toxicity	(PT)	II	[82]
*Alexandrium catenella* (Whedon et Kofoid) Balech	Saxitoxin, Gonyautoxin, c1-c4 toxins	(PT)	I, II	[82]
***Alexandrium insuetum***** Balech**	Unknown toxicity	(HB)	IV, V	[29,43]
*Alexandrium minutum* Halim	Gonyautoxins (1-4)	(PT)	I, II, IV, V	[43,64,83]
*Alexandrium tamarense* (Lebour) Balech	Gonyautoxins (1-4)	(PT)	I, II	[82,84]
*Alexandrium taylori* Balech	Gonyautoxin-4, Gonyautoxin-6	(PT)	I, II	[82]
*Amphidinium carterae* Hulburt	Maitotoxin	(PT)	IV, V	[29,85]
*Ceratium furca * (Ehrenberg) Claparède et Lachmann	Unknown toxicity	(PT)	I, II, III, IV, V	[29,73,78,80]
*Ceratium fusus *(Ehrenberg) Dujardin	Unknown toxicity	(PT)	I, II, III, IV, V	[29,73,78,80]
*Ceratium lineatum * (Ehrenberg) Cleve	Unknown toxicity	(PT)	IV, V	[29,79]
*Ceratium tripos* (Müller) Nitzsch	Unknown toxicity	(PT)	I, II, III, IV, V	[29,73,78,79,80]
*Coolia monotis* Meunier	Cooliatoxin	(PT)	I, III, IV	[79,86,87]
***Dinophysis acuminata * Claparède et Lachmann**	Okadaic acid, Dinophysistoxin-2	(TX)	I, II, IV	[42,43,71,85]
*Dinophysis acuta* Ehrenberg	Okadaic acid, Dinophysistoxin-2	(PT)	I	[88]
*Dinophysis caudata* Saville-Kent	Okadaic acid, Palytoxin	(PT)	I, II, IV, V	[29,42,43]
*Dinophysis fortii* Pavillard	Okadaic acid, Dinophysistoxin-1, Palytoxin	(PT)	I	[42]
*Dinophysis rotundata* Claparède et Lachmann	Dinophysistoxin-1	(PT)	I, IV	[42,79]
*Dinophysis sacculus* Stein	Okadaic acid	(PT)	I, II, III, IV, V	[29,43,73]
*Dinophysis tripos* Gourret	Dinophysistoxin-1	(PT)	I, II	[82,88]
*Diplopsalis lenticula* Bergh	Unknown toxicity	(PT)	I, V	[29,88]
*Gambierdiscus sp.*	Ciguatoxin, Maitotoxine	(PT)	III	[87]
*Gymnodinium catenatum* Graham	Gonyautoxins (1-4), Saxitoxin	(PT)	I	[84,88]
*Gyrodinium aureolum* Hulburt	1-acyl-3-digalactosyl glycerol, Octadeca- pentaenoic acid	(TX)	I, II	[46,88]
*Gyrodinium impudicum* Fraga et Bravo	Unkown toxicity	(PT)	I, IV	[79,84]
*Heterocapsa circularisquama* Horiguchi	hemolytic toxin2-a, hemolytic toxin 3-a	(PT)	V	[29]
***Karenia brevis*(*Gymnodinium breve*) (Davis) G. Hansen et Moestrup**	Brevetoxin-1, Brevetoxin-2, Brevetoxin-3	(TX)	I, II, III	[46,70,73,78]
*Karenia mikimotoi * (Miyake et Kominami ex Oda) Hansen et Moestrup	Gymnocin-A	(PT)	IV	[79]
*Karlodinium veneficum * (Ballantine) J. Larsen	Karlotoxin-1, Karlotoxin-2	(PT)	V	[29]
***Noctiluca scintillans *(Macartney) Kofoid et Swezy**	Unknown toxicity	(HB)	I	[43]
*Ostreopsis ovata* Fukuyo	Putative Palytoxin, Ovatoxin-a	(PT)	I, III, V	[29,86,87]
*Ostreopsis siamensis* Schmidt	Putative Palytoxin	(PT)	I, III	[86,87]
*Peridinium quinquecornen * Abé	Unknown toxicity	(PT)	V	[29]
*Prorocentrum arcuatum* Issel	Unknown toxicity	(PT)	V	[29]
*Prorocentrum borbonicum* Ten-Hage, Turquet, Quod, Puiseux-Dao et Couté	Borbotoxins	(PT)	I, III	[87,89]
*Prorocentrum dentatum* Stein	Unknown toxicity	(HB)	I, II	[46]
*Prorocentrum emarginatum* Fukuyo	Unknown toxicity	(PT)	I, III, IV	[79,87,89]
*Prorocentrum levis* M.A. Faust, Kibler, Vandersea, P.A. Tester & Litaker	Okadaic acid, Dinophysistoxin-2	(PT)	I	[89]
*Prorocentrum lima* (Ehrenberg) Stein	Okadaic acid, Dinophysistoxin-1, Dinophysistoxin-2	(PT)	I, II, III, V	[29,73,87,89]
***Prorocentrum micans* Ehrenberg**	Putative Palytoxin, Ovatoxin-a	(PT)	I, II, III, IV	[73,77,78,79]
***Prorocentrum minimum* (Pavillard) Schiller**	Unknown toxicity	(HB)	I, II, IV, V	[29,43,46]
***Prorocentrum obtusidens* Schiller**	Unknown toxicity	(HB)	I	[42,43]
***Prorocentrum redfeldii* Bursa**	Unknown toxicity	(HB)	I, IV	[43,79]
*Prorocentrum rhathymum* Loeblich III, Sherley et Schmidt	Okadaic acid	(PT)	I, III, IV	[85,87,89]
*Protoceratium reticulatum * (Claparède et Lachmann) Bütschli	Yessotoxin	(PT)	I	[84]
*Protoperidinium crassipes * (Kofoid) Balech	Azaspiracid toxin-1 Azaspiracid toxin-2 Azaspiracid toxin-3	(PT)	V	[29]
*Scrippsiella trochoidea* (Stein) Loeblich	Unknown toxicity	(HB)	I, II, III, IV, V	[29,46,73,78,79]
**Prymnesiophytes**				
***Phaeocystis pouchetii * (M.P. Hariot) G. Lagerheim**	Polyunsaturated aldehydes	(HB)	I, II, III	[46,62,73]
*Prymnesium parvum* N. Carter	Prymnesin-1, Prymnesin-2	(PT)	I, IV	[85,88]
**Rhaphidophytes**				
***Chattonella globosa* Y. Hara et Chihara**	Unknown toxicity	(HB)	I, IV	[42,43]
***Chattonella verucolosa* Y. Hara et Chihara**	Unknown toxicity	(HB)	I, IV	[42,43]
**Cyanobacteria**				
***Microcystis aeruginosa * (Kützing) Kützing**	Microcystin-LR	(TX)	II	[62]
***Lyngbya agardhii* P.L.Crouan & H.M.Crouan ex Gomont**	Unknown toxicity	(HB)	II	[62]
***Chroococcus gelatinosus* Geitler**	Unknown toxicity	(HB)	II	[62]
***Synechocystis sallensis* Skuja**	Unknown toxicity	(HB)	II	[62]
***Trichodesmium erythraeum* Ehrenberg**	Saxitoxin	(TX)	II	[62]

### 3.2. The ecological role of toxic, potentially toxic and bloom forming species in Greek coastal waters

In the present article (Table 1) we nominate toxic (TX) as the species producing blooms associated with evident toxic symptoms in the marine ecosystem, e.g., fish and shellfish death, or in humans consuming the poisoned fish and shellfish populations. Thus, consumption of contaminated shellfish by (a) the diatom *Pseudonitzschia seriata,* a domoic acid producer, caused [13] amnesic shellfish poisoning (ASP), (b) the dinoflagellate *Dinophysis sacculus,* an okadaic acid producer, caused [48] diarrhetic shellfish poisoning (DSP) and (c) the cyanobacterium *Microcystis aeruginosa*,a microcystin-LR producer, caused [61] extensive liver damage.

Potentially toxic (PT) are characterized as species carrying the toxigenic potential according to toxicological analyses, but their presence in an area has not been accompanied by toxic blooms and the relevant symptoms. A noticeable example is the toxic dinoflagellate (GTX1-4) *Alexandrium minutum*, whose presence did not produce toxic symptoms in the Greek coastal waters since their nutritional status did not favor blooms of this species [64].

Certain non-toxic species create high biomass (HB) blooms that have been characterized as harmful, because their occurrence produces discoloration of the water, undesirable aesthetic symptoms and anoxic harmful conditions to the ecosystem. They also cause severe economic problems due to loss to fisheries and tourism operations [65]. Massive growth of the dinoflagellates *Noctiluca *scintillans (late winter-early spring), *Chatonella globosa *(spring)and several species of the genus *Prorocentrum *in autumn *(P. micans*, *P. triestinum*, *P. obtusidens *and *P. rostratum) *caused severe water discoloration in Thermakos Gulf during the years 2000-2004 [43].

The total numbers of (TX), (PT) and (HB) algae reported in this work are 61 species. Dinoflagellates included 46 species contributing the 75% of total number (Table 1). Among them, three species are toxic (*Dinophysis acuminata, Gyrodinium aureolum, Karenia brevis*), seven species are forming high biomass (HB) harmful blooms and the rest (36) are potentially toxic species. Dinoflagellates are referred [66] as the group producing the most potent biotoxins known and with the largest number of HAB species, and the present data (75% dinoflagellates of total number of HAB species) are in accordance with this information.

Diatoms were represented by only six species-all potentially toxic-and this might be attributed to their nutrition requirements for a well balanced ratio (N:P:Si) of all nutrients. This necessity makes them poorer competitors than the non-siliceous dinoflagellates that seem to have a competitive advantage over diatoms if the stoichiometry of nutrients is deviated from its normal status in seawater [67]. 

Another advantage of dinoflagellates over diatoms is their nutritional mode, since several dinoflagellates are not exclusively phototrophic but heterotrophic/mixotrophic because they can shift to osmotrophy (uptake of dissolved organic substances) and/or phagotrophy (feeding on particulate organic carbon) under changes in nutrient supply ratios (N:P, C:P) and light-depleted conditions [8].

**Table 2:** Trophic strategies of heterotrophic HAB species.

Species	Feeding mechanism	Food type	Source
*Alexandrium catenella*	Osmotrophy	Urea, dextrans	[90]
*Alexandrium minutum*	Osmotrophy-Phagotrophy	Urea, Cyanobacteria	[91,92]
*Alexandrium tamarense*	Osmotrophy-Phagotrophy	Urea, Cyanobacteria, Cryptophytes	[92,93,94]
*Ceratium furca*	Phagotrophy	Ciliates	[95]
*Dinophysis acuminata*	Phagotrophy	Ciliates	[96]
*Gambierdiscus sp.*	Phagotrophy	Unknown pray	[8]
*Gymnodinium catenatum*	Phagotrophy	Cyanobacteria	[92]
*Gyrodinium impudicum*	Phagotrophy	Cyanobacteria, Algae	[94,97]
*Karenia brevis*	Osmotrophy-Phagotrophy	Urea, Cyanobacteria	[92,98]
*Karlodinium veneficum*	Osmotrophy-Phagotrophy	Urea, Cryptophytes	[99,100]
*Noctiluca scintillans*	Phagotrophy	Algae	[101]
*Ostreopsis ovata*	Phagotrophy	Unknown pray	[8]
*Ostreopsis siamensis*	Phagotrophy	Unknown pray	[8]
*Prorocentrum micans*	Phagotrophy	Cyanobacteria, Algae	[92,94]
*Prorocentrum minimum*	Osmotrophy-Phagotrophy	Urea, Cyanobacteria, Algae	[92,99,102]
*Protoperidinium crassipes*	Phagotrophy	Algae	[103]
*Scrippsiella trochoidea*	Phagotrophy	Cyanobacteria, Algae	[92,94]
*Prymnesium parvum*	Phagotrophy	Algae	[104]
*Microcystis aeruginosa*	Osmotrophy	Leucine	[69]

The 19 identified mixotrophic species in this investigation (Table 2) included 17 dinoflagellates, one prymnesiophyte and one cyanobacterium. Mixotrophic dinoflagellates comprised 40% of the total (46) species in the Dinophyceae class (Table 1) and their feeding types are well known. Nine mixotrophic species (*Ceratium furca*, *Dinophysis acuminata*, *Gymnodinium catenatum*, *Gyrodinium impudicum*, *Noctiluca scintillans*, *Prorocentrummicans*, *Protoperidinium crassipes*, *Scrippsiella trochoidea*, *Prymnesium parvum)* have been reported as phagotrophic, having the ability to feed on prokaryote prey (e.g., cyanobacteria) and/or eukaryote algae (dinoflagellates, cryptophytes). However, the prey of phagotrophic *Gambierdiscus sp.*, *Ostreopsis ovata*, *O. siamensis*, is unknown. For species supplementing their nutrition with osmotrophy (*Alexandriumcatenella*) or osmotrophy and phagotrophy (*Alexandrium minutum*, *A. tamarence*, *Karenia brevis*, *Karlodinium veneficum*, *Prorocentrum minimum*), urea proved to be an important nitrogen source, with the exception of the cyanobacterium *Microcystis aeruginosa*, which may utilize leukine.

Among the 61 species presented in Table 1, certain algae (16) have been associated with the occurrence of important HAB incidents in the investigated areas during the last 30 years, and six among these are heterotrophic species. Table 3 presents the seasonal and spatial distribution of the HAB incidents and the associated impact in the biotic community and water quality.

**Table 3:** Important HAB incidents in Greek coastal waters.

Species	Season/year of max. abundance (Cells.L^−1^)	Gulf	Impact	Source
***Alexandrium insuetum ***	April 2003 (2.5 × 10^6^ )	Amvrakikos	Water discoloration	[43]
	May 2004 (4.7 × 10^5^)			
***Dinophysis acuminata***	Jan. 2000 (8.5 × 10^4^)	Thermaikos	Diarrhetic shellfish toxins	[42,71]
	Feb. 2002 (3.7 × 10^4^)			
	March 2003 (2.2 × 10^3^)			
	May 2004 (1.1 × 10^4^ )			
***Karenia brevis ***	Sept. 1977 (1.0 × 10^7^)	Saronikos	Massive fish kill	[70,105]
	Sept. 1978 (5.0 × 10^7^)			
	Oct. 1987 (2.7 × 10^7^)			
***Noctiluca scintillans***	February-March 2000-2004 (>1.0 × 106)	Thermaikos	Water discoloration	[43]
	March 1978 (1.1 × 10^5^)	Kavalas	Water discoloration	[105]
***Prorocentrum micans ***	April 1994 (3.7 × 10^7^)	Thermaikos	Water discoloration Water discoloration	[43]
	May 1993 (1.1 × 10^6^)	Saronikos		[46]
***Prorocentrum minimum***	April 2003 (1.2 × 10^5^)	N. Aegean coastal area, Saronikos, Amvrakikos	Water discoloration	[43]
	April 2003 (1.1 × 10^5^)			
	Autumn 2003 (1.0 × 10^5^)			
***Prorocentrum obtusidens***	Jan. 2000 (1.2 × 10^6^)	Thermaikos	Water discoloration	[43]
	Jan. 2001 (1.2 × 10^6^)			
***Prorocentrum redfeldii ***	Winter 2000 (1.2 × 10^6)^	Thermaikos	Water discoloration	[43]
	Winter 2001 (6.0 × 10^6)^			
***Phaeocystis pouchetii ***	March 1989 (2.5 × 10^6)^	Saronikos	Water discoloration	[46]
	August 1993 (3.5 × 10^7)^	Evoikos	Mucilage	[62]
	Sept. 1999 (2.7 × 10^6)^			
***Chattonella globosa***	Spring 2001 (>10^4^)	Thermaikos	Water discoloration	[43]
	Spring 2002 (>10^4^)			
	Spring 2003 (>10^4^)			
***Chattonella verucolosa ***	Dec. 1998 (Massive presence)	Amvrakikos	Mass finfish mortality	[43]
***Microcystis aeruginosa***	Sept. 1999 (9.9 × 10^5^)	Evoikos	Mucilage	[62]
***Lyngbya agardhii***	Sept. 1999 (4.8 × 10^3^ filaments.L^−1^)	Evoikos	Mucilage	[62]
***Chroococcus gelatinosus***	Sept. 1999 (8.2 × 10^5^)	Evoikos	Mucilage	[62]
***Synechocystis sallensis***	Sept. 1999 (8.9 × 10^4^)	Evoikos	Mucilage	[62]
***Trichodesmium erythraeum***	Sept. 1999 (7.1 × 10^4^ trichomes.L^−1^)	Evoikos	Mucilage	[62]

The present data demonstrate that HAB episodes in Greek coastal waters are sporadic in time, space and recurrence of the causative species. Blooms (up to 5.0 × 10^7^ cells.L^−1^) of *Karenia brevis (Gymnodinium breve)* were recorded only in the Saronikos Gulf, three times (September 1977, September 1978, and October 1987) with massive fish kill. Outbreaks of *Dinophysis acuminata* (up to 8.5 × 10^4^ cells.L^−1^) were recorded only in the Thermaikos Gulf in January 2000, April 2001, February 2002, March 2003 and May 2004, and they were associated with extensive shellfish deaths. However, this species was also observed in the Amvrakikos and Malliakos Gulfs at several times in low abundances and without toxic symptoms. The huge growth (5.4 × 10^6^cells.L^−1^) of *Noctiluca scintillans* caused water discoloration in late winter-early spring occasionally during 2000-2004 in Thermaikos and in Kavala Gulfs. The outbursts of four species of the genus *Prorocentrum *were also associated with water discoloration. *P. obtusidens*, *P. redfeldii* and *P. micans* occurred in the Thermaikos Gulf during the winter 2000-2001 at abundances up to 6.0 × 10^6^ cells.L^−1^ and *P. minimum* was recorded (up to 1.2 × 10^5^ cells.L^−1^) in April 2003 along the N. Aegean coastal line and in the Saronikos Gulf, and in autumn 2003 in the Amvrakikos Gulf. However, the presence of *P. minimum* in the Kalloni Gulf did not cause any undesirable incidents [29]. Mass occurrence (4.7 × 10^5^-2.5 × 10^6^ cells.L^−1^) of *Alexandrium insuetum* caused water discoloration in the Amvrakikos Gulf in the spring of 2003 and 2004, but in Kalloni Gulf did not create harmful effects [29].

The two Rhaphidophyte species of Chattonella were also involved in severe HAB phenomena. The species C. globosa grew massively (>104 cells.L−1), causing water discoloration during spring 2001-2003 in the Thermaikos Gulf, whereas considerable growth of C. veruculosa caused finfish mortality in the Amvrakikos Gulf in December 1998. The Prymnesiophyte Phaeocystis pouchetii, growing at concentrations up to 3.5 × 107 cells.L−1, caused water discoloration in the Saronikos Gulf (March 1989, August 1993) and “mucilage” problems in the Evoikos Gulf (September 1999). In September 1999, the co-occurrence of five species of the cyanophyceae, Microcystis aeruginosa (9.9 × 105 cells.L−1), Lyngbya agardhii (4.8 × 103 filaments.L−1), Chroococcus gelatinosus (8.2 × 105 cells.L−1), Synechocystis sallensis (8.9 × 104 cells.L−1) and *Trichodesmium erythraeum* (7.1 × 104 trichomes.L−1) produced a serious harmful bloom in the Evoikos Gulf. The sea surface was covered by mucus-forming “blankets” and “marine snow” transported horizontally and vertically and causing problems to recreation, public health and fish harvesting. 

From the ecological point of view, most (TX), (PT) and (HB) algae (Table 1) are “normal” components of inshore waters [72,73]. However, major gaps still exist in our understanding of the factors triggering only certain species to initiate and develop harmful populations. There is evidence that HABs are eutrophication-induced phenomena thriven by anthropogenic activities. Records on the trophic status of the Aegean and Ionian Gulfs [1] proved that the investigated areas (Figure 1) were characterized “eutrophic” because the chl α concentrations were higher (>>1.0 mg chlα. m^−3^) in relation to the values (<<0.5 mg chlα. m^−3^) prevailing in the oligotrophic open oceanic waters [74]. The information available on the eutrophication-HAB relationship has recently increased, regarding the general explanation of the competition of phytoplankton species in relation to overall nutrient availability and the ratio between different nutrient species [65].

It is interesting to notice that the species *Alexandrium insuetum*, *A. tamarense*, *Gymnodinium catenatum*, *Gyrodinium aureolum*, *Coolia monotis*, *Ostreopsis ovata *and *O. siamensis* are not indigenous, but alien species of the Mediterranean Sea. They have been introduced via ship traffic for the Atlantic, Pacific and Indian Oceans [75] and it is obvious that the “ballast water” problem needs urgent attention [76].

## 4. Conclusions

The available data indicate that 61 identified HAB species (toxic, potentially toxic and high biomass producing algae) have spread across the Greek coastline during the last 30 years. Among these, certain algae (16) were associated with the occurrence of important HAB incidents causing damage in the marine biota and the water quality. There is a strong indication that these incidents were eutrophication-induced phenomena, but sporadic in time, space and recurrence of the causative species.
